# High-Sensitivity Ultrasonic Guided Wave Monitoring of Pipe Defects Using Adaptive Principal Component Analysis

**DOI:** 10.3390/s21196640

**Published:** 2021-10-06

**Authors:** Junwang Ma, Zhifeng Tang, Fuzai Lv, Changqun Yang, Weixu Liu, Yinfei Zheng, Yang Zheng

**Affiliations:** 1 College of Biomedical Engineering and Instrument Science, Zhejiang University, Hangzhou 310027, China; 22015061@zju.edu.cn (J.M.); 11815033@zju.edu.cn (W.L.); zyfnjupt@zju.edu.cn (Y.Z.); 2 State Key Laboratory of Fluid Power and Mechatronic Systems, School of Mechanical Engineering, Zhejiang University, 38 Zheda Road, Hangzhou 310027, China; lfzlfz@zju.edu.cn; 3 South China Branch of National Oil & Gas Piping Network Corporation, Guangzhou 510180, China; 22115081@zju.edu.cn; 4 Research Center for Intelligent Sensing, Zhejiang Lab, Hangzhou 311100, China; 5 China Special Equipment Inspection and Research Institute, Beijing 100029, China; zhengyang@csei.org.cn

**Keywords:** pipe, ultrasonic guided wave monitoring, high-sensitivity defect identification, adaptive principal component analysis, nondestructive evaluation

## Abstract

Ultrasonic guided wave monitoring is regularly used for monitoring the structural health of industrial pipes, but small defects are difficult to identify owing to the influence of the environment and pipe structure on the guided wave signal. In this paper, a high-sensitivity monitoring algorithm based on adaptive principal component analysis (APCA) for defects of pipes is proposed, which calculates the sensitivity index of the signals and optimizes the process of selecting principal components in principal component analysis (PCA). Furthermore, we established a comprehensive damage index (K) by extracting the subspace features of signals to display the existence of defects intuitively. The damage monitoring algorithm was tested by the dataset collected from several pipe types, and the experimental results show that the APCA method can monitor the hole defect of 0.075% cross section loss ratio (SLR) on the straight pipe, 0.15% SLR on the spiral pipe, and 0.18% SLR on the bent pipe, which is superior to conventional methods such as optimal baseline subtraction (OBS) and average Euclidean distance (AED). The results of the damage index curve obtained by the algorithm clearly showed the change trend of defects; moreover, the contribution rate of the K index roughly showed the location of the defects.

## 1. Introduction

With the increase in the service life of oil and gas pipes, various kinds of defects or damage will occur gradually. The ultrasonic guided wave testing technique [1,2,3,4], with its advantages of long propagation distance, low attenuation, and large detection range compared with the conventional methods (such as magnetic flux leakage, eddy current, and X-ray [5,6,7]), has thus been widely used in structural health monitoring (SHM) and nondestructive testing (NDT) for pipe structures.

During testing for pipe defects, monitoring by ultrasonic guided waves has a higher signal-to-noise ratio (SNR) and sensitivity compared with detection [8,9]. The transducer is fixed permanently to reduce the manual operation and the environmental noise. By comparing the real-time measurement signal with the original signal, the defect of a pipe can be inspected more intuitively [10,11,12]. However, the sensitive of the commercial guided wave system is only 0.6% SLR [13]. In a previous study, Rose verified the possibility of detecting a pipe with 0.36% SLR in simulation, and the monitoring performance was found to be better [14], and Jacob [15] also found in the experiment that the hole defect of 0.25% SLR can be monitored on the straight pipe, but if the hole defect is produced after a bend region of the bent pipe, it can hardly be monitored. There are two main difficulties in the micro defect monitoring of pipes:

1. In the continuous monitoring of a pipe, there is still a probability that the instrument will produce large noise while working normally;
2. Slight differences in the monitoring signals exist at different times of the day, and due to the influence of temperature on the materials, the guided wave propagation is affected too [16].

The traditional solution to these problems is to increase the excitation power and transducer coupling efficiency at the instrument level and to establish the temperature compensation at the signal preprocessing level [17]. However, many neglected factors that are difficult to check also limit the monitoring performance.

During monitoring signal processing research, the pipe signal recorded by an instrument, in which the temperature influence has been compensated by optimal time-domain stretch method [18], is used in various defect evaluation algorithms. For example, the original signal is subtracted from the test signal after Hilbert envelope processing to identify the defects, which is a method called optimal baseline subtraction (OBS) [19]. In later research, by calculating the Euclidean distance between the original signals and the test signals, a boundary distance was set to assess the defects, called the average Euclidean distance method (AED) [20,21], which also improves monitoring sensitivity. Although the above methods are commonly used, the calculation information is redundant, and some useless points in the collected signal will interfere with the results.

Feature space decomposition reduction technology has been found to have great potential in enhancing the representativeness of data [22]. Principal component analysis (PCA), which uses this technology, is a commonly used data dimension reduction method [23,24]. It aims to find the principal components related to the main features in the data to represent the original signals, so it is less affected by random noise caused by the environment. Independent component analysis (ICA) is also used to decompose the feature space [25]. It is simple to extract effective signals from complex signals, but the extracted components are independent, which makes it difficult to separate the noise signals from a Gaussian distribution.

In order to improve the sensitivity of detecting small defects, the adaptive principal component analysis algorithm (APCA) is proposed in this paper. Based on the traditional PCA, an adaptive link is added to the selection process of the principal component to enhance the sensitivity of the monitoring algorithm. In Section 2, we introduce the basic preprocessing of signals and the principle of APCA for defect identification. Section 3 shows the performance of the APCA algorithm for straight pipes, bent pipes, and spiral pipes, as well as a comparison of APCA with OBS and AED. Conclusions follow in Section 4.

## 2. Signal Processing Methods

### 2.1. Pre-Processing

**Figure 1:**
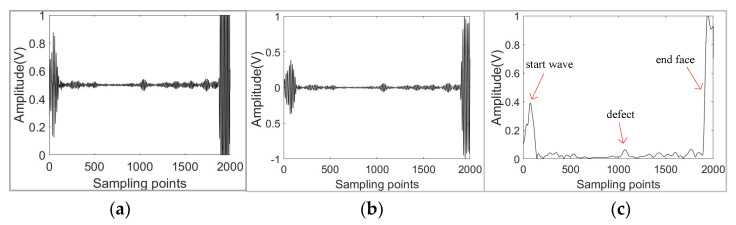
The guided wave signal of straight pipe with hole defect (0.45% SLR). (**a**) Instrument signal; (**b**) Filtered signal; (**c**) Envelope signal.

For the guided wave signal collected by the instrument, due to the noise caused by the instrument or the transducer, the guided wave signal is usually preprocessed before the application of the monitoring algorithm to identify the defect. It mainly includes Butterworth data filtering and Hilbert envelope extraction, the application of both of which has been proved to improve the signal-to-noise ratio and to reduce the influence of the end surface and weld [26,27]. The Hilbert envelope of the filtered signal was obtained after removing the bias, which can be written as the following Equations (1) and (2):

(1) $$\hat{x}\left(t\right)\;=\;\tilde{x}\left(t\right)\;/\sqrt{\frac{1}{n}\sum_{i=1}^{n}\tilde{x}{\left(i\right)}^{2}}$$

(2) $$x_{e}\left(t\right)\;=\;\sqrt{\hat{x}{\left(t\right)}^{2}+\left(\frac{1}{\pi }\int \frac{\hat{x}\left(t-\tau \right)}{\tau }d\tau \right)}$$

where $\tilde{x}\left(t\right)$ is the signal after filtering and removing the bias (Figure 1b). An example of the Hilbert envelope of guided wave signals ($x_{e}\left(t\right)$) in our experiment is exhibited in Figure 1c. It can be seen that the envelope signal had more prominent defect characteristics than the instrument signal (Figure 1a).

In the first stage, we collected original signals on the undamaged pipe and carried out the above preprocess. In the second stage, the test signals were collected on pipe with different cross section loss ratio (SLR) of hole defects; the SLR refers to the ratio of defect area to the cross section of pipe, and it is used to measure the degree of defect and can be expressed as the following Equation (3):

(3) $$\mathrm{SLR}=\frac{d*h}{\pi *D*T}$$

where d is the diameter of hole defect, h is the depth of holing, D is the diameter of pipe, and *T* is the thickness of pipe. In addition to that, the original signals and test signals are also temperature compensated to avoid the interference of temperature; this compensation method [28] can refer to the previous research of our laboratory. The signal processing conditions and transducer installation conditions of the two stages are the same, which also lays the foundation for the implementation of the later monitoring algorithm.

### 2.2. Feature Decomposition

Suppose that the number of collected original signals is *m* and that each signal contains *n* sampling points and is regarded as a vector $x_{i}$; then, the original signals matrix can be expressed as Equation (4):

(4) $$X_{n\times m}=[x_{1}\;\;\;x_{2}\;\;\;\ldots \;\;\;x_{m}]=\left[\begin{array}{cc} \begin{array}{ccc} x_{11} & x_{12} & \cdots \\ x_{21} & x_{22} & \cdots \end{array} & \begin{array}{c} x_{1m}\\ x_{2m} \end{array}\\ \begin{array}{ccc} \vdots & \vdots & \ddots \\ x_{n1} & x_{n2} & \cdots \end{array} & \begin{array}{c} \vdots \\ x_{nm} \end{array} \end{array}\right]$$

First, an *n*-order covariance matrix C is constructed for the $X_{n\times m}$, and the eigenvalues and eigenvectors of the covariance matrix C are also calculated, which can be expressed as Equations (5) and (6):

(5) $$C=\frac{1}{n}\sum_{i}^{n}\left(x_{i}-\bar{x}\right){\left(x_{i}-\bar{x}\right)}^{T}$$

(6) $$USU^{T}=C$$

The matrix *U* obtained by the operation is an *n* × *n* characteristic matrix, and the matrix S is a diagonal matrix composed of eigenvalues $\lambda_{1},\;\lambda_{2}\ldots \lambda_{n}$; the eigenvalues are sorted from large to small in matrix S. Selecting the column of matrix *U* as the vector, we will get *n* eigenvectors $u_{1}$, $u_{2}$,$\;u_{3}\;$…$\;u_{n}$. Then, the dimension-reduced *k*-dimensional matrix $Y_{k\times m}$ can be obtained by the following Equation (7):

(7) $$Y_{k\times m}=\left[\begin{array}{cc} \begin{array}{ccc} y_{11} & y_{12} & \cdots \\ y_{21} & y_{22} & \cdots \end{array} & \begin{array}{c} y_{1m}\\ y_{2m} \end{array}\\ \begin{array}{ccc} \vdots & \vdots & \ddots \\ y_{k1} & y_{k2} & \cdots \end{array} & \begin{array}{c} \vdots \\ y_{km} \end{array} \end{array}\right]=U_{k}^{T}\cdot X_{n\times m}$$

where $U_{k}^{\;}$ is taken from the first *k* vectors of matrix U, which is also called the principal component matrix. The subspace supported by it is called the principal component subspace (PCS), which is used to measure the fitting degree of test signals and original signals;$\;Y_{k\times m}$ is the projection of the signals on the PCS, the greater the distribution difference, the greater the projection value. $U_{n-k}^{\;}$ is taken from the last *n-k* vectors of matrix *U*, and it also supports the residual subspace (RS), which is used to measure the residual between the test signals and the principal component model. Then, the damage evaluation index should be established, which is based on the above two subspaces.

According to the previous discussion, the PCA mapped the data into PCS and RS, and then two statistics were introduced—Hotelling $T^{2}$ ($T^{2}$) and the squared prediction error (SPE) [29,30]—to monitor the occurrence of defect.

$T^{2}$ was used to measure the information size and change range in the PCS. It is defined as the following Equations (8) and (9):

(8) $$T^{2}=x_{i}{}^{T}U_{k}^{\;}\;S_{k}{}^{-1\;}U_{k}^{T}\;x_{i}\;<\;T_{a}$$

(9) $$T_{a}=\frac{k\left(n^{2}-1\right)}{n\left(n-k\right)}{\;F}_{a}\left(k,n-k\right)$$

where $F_{a}$ is the F distribution of statistics,${\;S}_{k}$ is a diagonal matrix composed by first *k* eigenvalues $\lambda_{1},{\;\lambda }_{2}\ldots \lambda_{k}$ of *C*. *a* is the confidence level (generally 99%), and $T_{a}$ is the control limit of $T^{2}$.

The SPE was used to measure the deviation size of the test signal in the RS. It is defined as the following Equations (10) and (11):

(10) $$\mathrm{SPE}=||U_{n-k}^{T}\cdot x_{i}||{\;}^{2}<\;Q_{a}$$

(11) $$Q_{a}=\theta_{1}{\left[\frac{C_{a}h_{0}\sqrt{2\theta_{2}}}{\theta_{1}}+1+\frac{\theta_{2}h_{0}\left(h_{0}-1\right)}{\theta_{1}^{2}}\right]}^{1/h_{0}}$$

In this formula, $C_{a}$ is the confidence limit of the standard normal distribution, $C_{a}$ is the confidence level (generally 99%), and $Q_{a}$ is the control limit of the SPE. θ and $h_{0}$ are performed as Equations (12) and (13):

(12) $$\theta_{i}=\overset{n}{\underset{j=k+1}{\sum }}\lambda_{j}^{i}$$

(13) $$h_{0}=1-\frac{2\theta_{1}\theta_{3}}{3\theta_{2}^{2}}$$

Under normal conditions, the projection of the original signals in RS should be very small. When the damage occurs in the pipe, the observation sample will deviate from the PCS and increase its projection in RS, the information size and deviation size will change significantly, and the $T^{2}$ and SPE value will exceed their limits. In order to avoid the influence of instrument noise and to reduce the false alarm rate, we considered that only when $T^{2}$ > $T_{a}$ and SPE > $Q_{a}$ can the occurrence conditions of defects be satisfied.

### 2.3. Adaptive Principal Component Analysis

When PCA is used to construct the principal component matrix, the number of principal components must be determined, which will directly affect the performance of defect monitoring and diagnosis. If the number of principal components is too small, the PCS contains too little information, which makes the $T_{a}$ too small; moreover, the RS will also contain redundant information, and this can easily lead to false detection. If the number of principal components is too high, the large amount of useless information in the PCS may submerge the defect information, which makes it difficult to detect small defects [31,32]. The most widely used method of principal component selection is the cumulative percentage variance (CPV) method [33]. When the first *k* principal components are selected, the CPV is obtained by Equation (14):

(14) $$\mathrm{CPV}=\sum_{i}^{k}\lambda_{i}/\sum_{i}^{n}\lambda_{i}$$

Generally, CPV ∈ (0.85, 0.95) is used to balance principal component information and dimension reduction points [34]. However, the CPV is selected by people subjectively, and it is difficult to meet the diagnosis requirements of small defects. In order to link the construction of feature subspace with diagnosis of defects, this paper proposes the APCA method to adaptively select the number of principal components according to the statistical index $T^{2},\;SPE,$ and their control limits $T_{a},Q_{a}$.

To begin with, for the original signals without defects and test signals with defects, in previous Section 2.2, we explained that $T^{2}$ will exceed $T_{a}$ when the defects are detected; the damage index can be given by Equation (15):

(15) $$DI_{1}=\frac{T^{2}{}_{test}}{T_{a}}$$

where the $T^{2}{}_{test}$ means the $T^{2}$ index of a test signal. Therefore, we can consider that the defects are detected when the damage index $DI_{1}$ > 1; the larger the damage index is, the easier it is to find defects. However, the establishment of the principal component model depends on the original signals without defects, so we define a sensitivity index, which can be expressed by Equation (16):

(16) $$SI_{1}=\frac{T^{2}{}_{original}}{T_{a}}$$

where the $T^{2}{}_{original}$ means the $T^{2}$ index of original signals. The larger sensitivity index means that the threshold $T_{a}$ of defects is easier to achieve and that the detection of defects will become more sensitive. Next, we will rewrite the Equation (16) for experimental signals.

**Figure 2:**
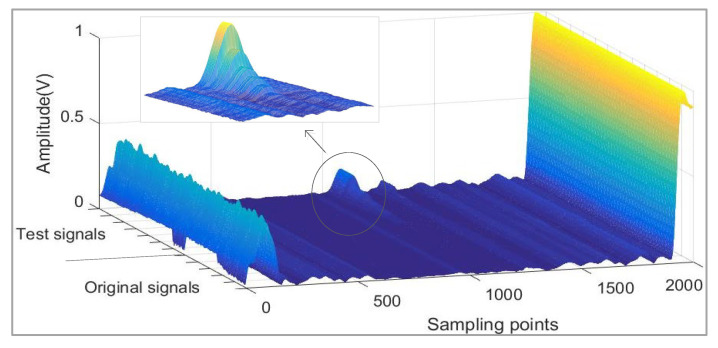
The dataset of signals in straight pipe experiment.

The process of this method is demonstrated with our experimental signals. The example used was a straight pipe with a defect at 1 m. The signals of this pipe include 100 sets of original signals without defects and 140 sets of test signals with different defects (0%, 0.075%, 0.15%, 0.225%, 0.3%, 0.45%, and 0.6% SLR; see Table 1 for details). The dataset of signals is also shown in Figure 2.

For the original signals, an original sensitivity index is established in PCS by Equation (17):

(17) $$OSI_{1}=\frac{1}{N_{original}}\overset{N_{original}}{\underset{i=1}{\sum }}\frac{T^{2}{}_{i}}{T_{a}}$$

where the $T^{2}{}_{i}$ means the $T^{2}$ index of original signals. Similar processing is also carried out in the RS; the damage index and original sensitivity index are obtained by Equations (18) and (19):

(18) $$DI_{2}=\frac{{\mathrm{SPE}}_{test}}{Q_{a}}$$

(19) $$OSI_{2}=\frac{1}{N_{original}}\overset{N_{original}}{\underset{i=1}{\sum }}\frac{SPE_{i}}{Q_{a}}$$

A comprehensive original sensitive index considering PCS and RS is defined by Equation (20):

(20) $$OSI_{\;}=\frac{OSI_{1}}{2}+\frac{OSI_{2}}{2}\;$$

**Figure 3:**
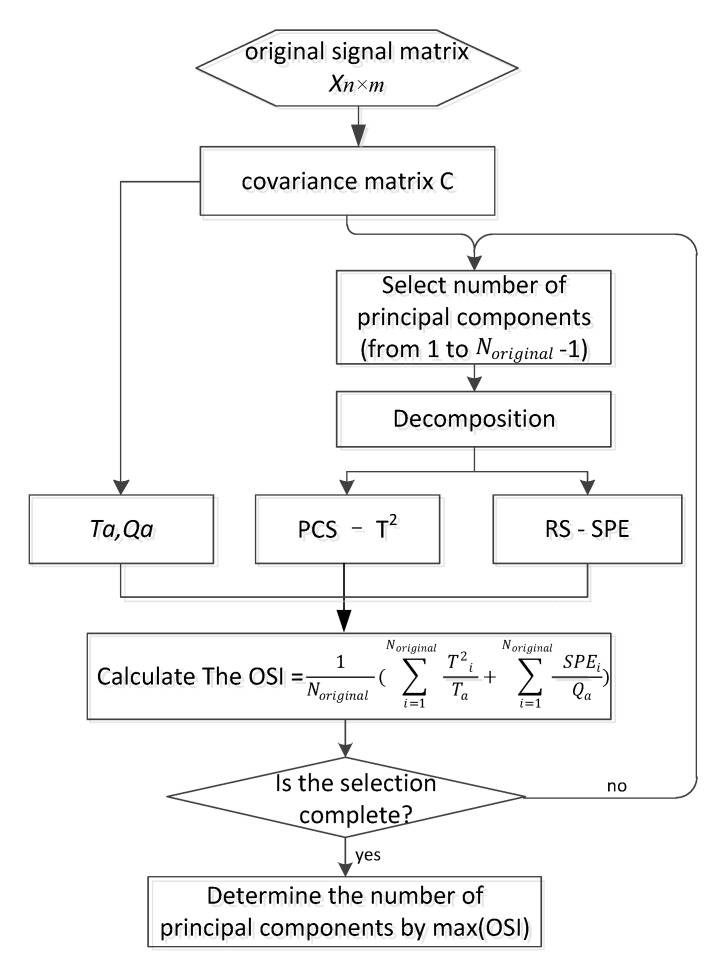
Flow diagram of the APCA method.

We can achieve a better sensitivity by referring to OSI when selecting the principal components. The processing flow of this adaptive method can be seen intuitively in Figure 3.

**Figure 4:**
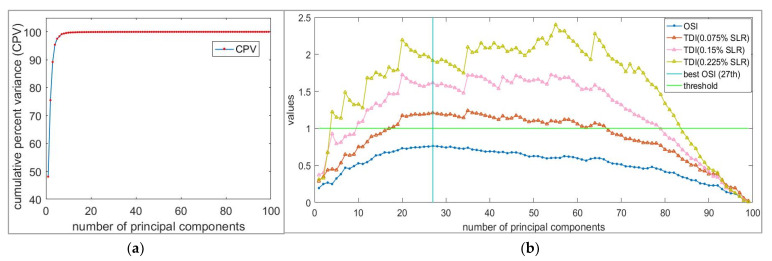
Influence of different number of principal components. (**a**) Influence on cumulative percentage variance (CPV); (**b**) Influence on original sensitive index (OSI) and test damage index (TDI).

The selection results of principal components in this dataset are shown in Figure 4b; a comprehensive test damage index used to verify the selection effect is defined by Equation (21):

(21) $$TDI_{\;}=\frac{DI_{1}}{2}+\frac{DI_{2}}{2}\;$$

Obviously, a larger *TDI* means a better recognition effect of the APCA method. Therefore, we studied the relationship between the number of principal components, TDI, OSI, and CPV, as shown in Figure 4.

Figure 4a shows the influence of different principal components on CPV; it can be seen that the CPV of the first five principal components has exceeded 95%. In Figure 4b, we can find that with the increase in principal component number, both the OSI and TDI increase first and then decrease. Obviously, the five principal components selected by CPV cannot meet the needs of defect diagnosis. When the defect is small (0.075% SLR), the trend of the OSI and TDI is relatively similar. This trend will change only when the defect becomes larger (0.15% SLR). We can find that when the first 27 principal components are selected according to OSI, the first 27 principal components also have a good recognition effect for the defects of 0.075% SLR.

**Figure 5:**
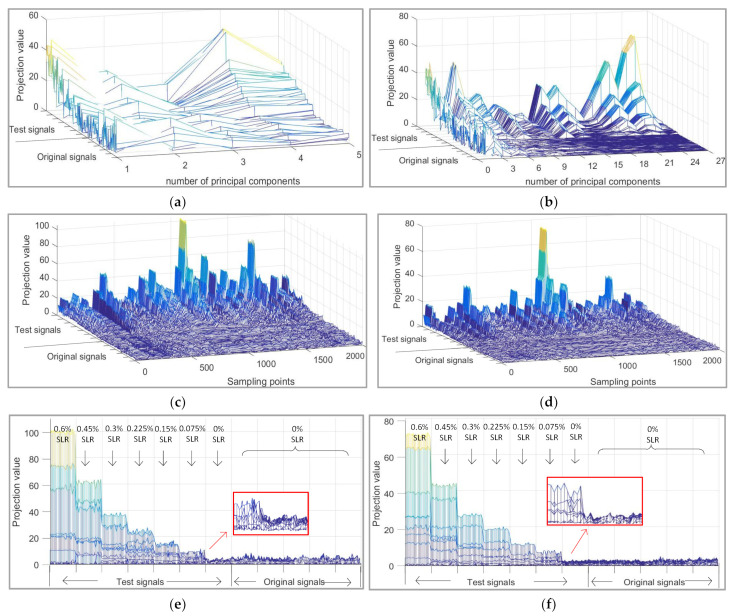
Comparison of feature decomposition effects between principal component analysis (PCA) and adaptive principal component analysis (APCA). (**a**) Projection value of PCA in principal component subspace (PCS); (**b**) Projection value of APCA in PCS; (**c**) Projection value of PCA in residual subspace (RS); (**d**) Projection value of APCA in RS; (**e**) Projection value of APCA in RS (the side view of (**c**)); (**f**) Projection value of APCA in RS (the side view of (**d**)).

The feature extraction effect of signals was also compared with different numbers of principal components in Figure 5 (5 principal components (95% CPV) selected subjectively and 27 principal components selected adaptively by our method).

As shown in Figure 5a,b, when the original signals are projected into PCS, the damage trend becomes more clear and easier to identify. However, this trend is more obvious in the 12th to 27th principal components; the first 12 principal components do not record the change trend of defects well. The principal component information selected subjectively through CPV is too little to cover the change of defects. It also proves that a more appropriate number of principal components are selected by the APCA method.

As seen in Figure 5c,d, when the original signals are projected into RS, the damage location becomes more clear and easier to identify. However, too few principal components are selected by CPV, which leads to a redundancy of residual information. By observing the side view of projection information in RS (Figure 5e,f), we can find that the defects are separated better in the RS through our method.

**Figure 6:**
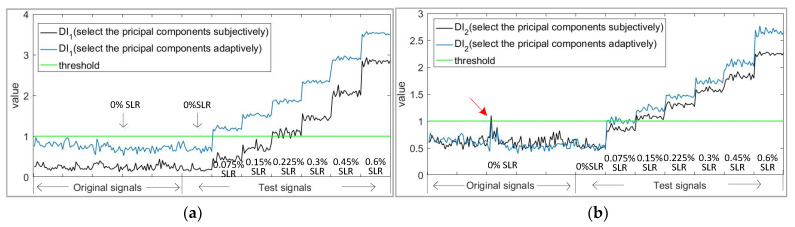
Comparison of damage index between principal component analysis (PCA) and adaptive principal component analysis (APCA). (**a**) Damage index ($DI_{1}$) of principal component subspace; (**b**) Damage index ($DI_{2}$) of residual subspace.

At the same time, the damage indexes $DI_{1}$ and $DI_{2}$ were also calculated to reflect the damage detection sensitivity. The results obtained by selecting the principal components by different methods are compared in Figure 6.

It can be seen from Figure 6 that the damage index of APCA is higher than that of traditional PCA on different kinds of defects. At the same time, the above discussion also proves that the APCA method, which selects the principal component by the sensitive index adaptively, has the effect of greatly improving the PCA algorithm.

### 2.4. Post-Processing

During the sampling process of the instrument, a very small part of the signals may be disturbed by electromagnetic noise, which are defined as outlier samples. The useless residual information of outlier samples will cause the rise of *Qa*, and thus will lead to an insensitive damage index. As can be seen from the previous Figure 6b, the red arrow points to an outlier sample.

In this step, the Pauta Criterion [35] is used to screen the outlier samples in the RS, which is expressed in Equations (22) and (23):

(22) $$SPE\left(i\right)\in \bar{SPE}\pm \;3\times \sigma \left(SPE\right)$$

(23) $$\sigma \left(SPE\right)=\sqrt{\frac{\sum_{i=1}^{N_{original}}{\left(SPE\left(i\right)-\bar{SPE}\right)}^{2}}{N_{original}}}$$

**Figure 7:**
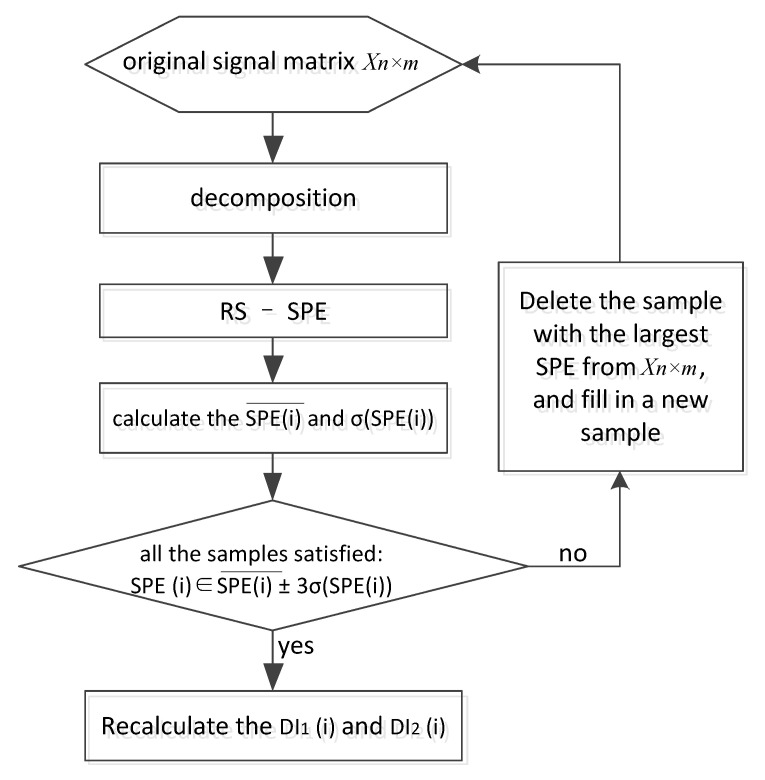
Flow diagram of removing outlier samples.

When the $SPE\left(i\right)$ of original signal does not satisfy the Equation (22), we consider it to be an outlier sample. The process of screening outlier samples can be shown in Figure 7.

**Figure 8:**
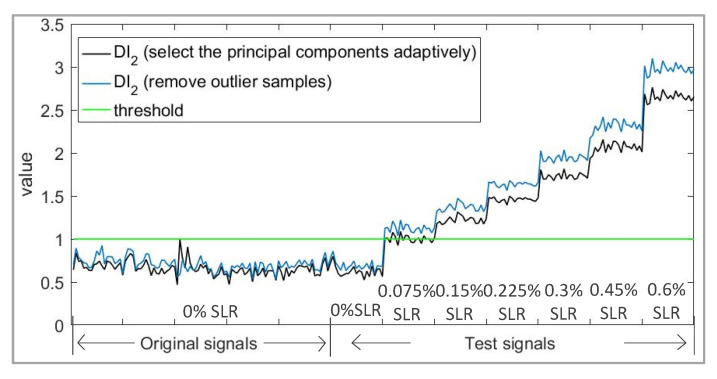
Comparison of damage index ($DI_{2}$) before and after removing outlier samples.

After this processing, the SPE become lower, and the damage index $DI_{2}$ becomes more sensitive to defects, as is shown in Figure 8.

### 2.5. Damage Judgment

**Figure 9:**
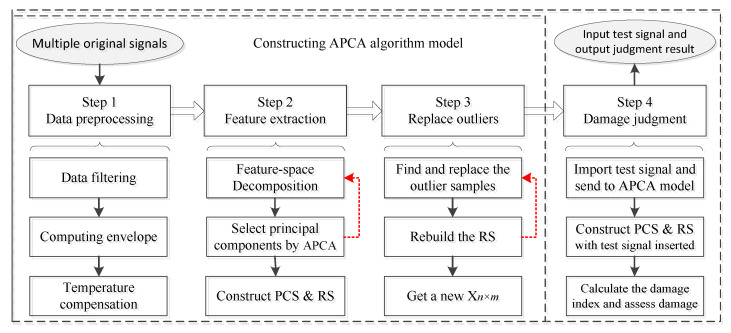
Flowchart of signal acquisition and defect monitoring.

As shown in Figure 9, after the signal processing in Section 2.1, Section 2.2, Section 2.3 and Section 2.4, the steps 1, 2, and 3 needed to build the APCA model have been satisfied. In order to judge the test signal (step 4), we needed to establish a comprehensive damage index to diagnose the defects.

In step 4, after the latest test signal is collected, consider it as a vector $x_{s}$ with n points, and insert it into the original signals matrix $X_{n\times m}$ for operation. The selection of the principal component was determined during the construction of the APCA model; the operation can be performed as Equation (24):

(24) $$Y_{k\times \left(m+1\right)}^{\prime }=\left[\begin{array}{cc} \begin{array}{ccc} y_{11} & y_{12} & \cdots \\ y_{21} & y_{22} & \cdots \end{array} & \begin{array}{c} y_{1m}y_{1s}\\ y_{2m}y_{2s} \end{array}\\ \begin{array}{ccc} \vdots & \vdots & \ddots \\ y_{k1} & y_{k2} & \cdots \end{array} & \begin{array}{c} \vdots \vdots \\ y_{km}y_{ks} \end{array} \end{array}\right]=U_{k}^{T}\cdot [x_{1}\;\;\;x_{2}\;\;\;\ldots \;\;\;x_{m}\;\;x_{s}]$$

where $Y_{k\times \left(m+1\right)}^{\prime }$ represents the characteristic matrix and the low-dimensional principal component matrix obtained by adding the test signal. The *SPE* and $T^{2}$ values of $x_{s}$ can be calculated by Equations (8) and (10).

In order to avoid the influence of a single index on the results, the comprehensive damage index K is defined by Equation (25):

(25) $$K=\left\{\begin{array}{cc} DI_{1} & \left(DI_{1}<1\right)\\ DI_{2} & \left(DI_{2}<1\right)\\ TDI & \left(DI_{1},DI_{2}>1\;or\;DI_{1},DI_{2}<1\right) \end{array}\right.$$

where the K is the comprehensive damage index of the APCA method for monitoring defects. When $DI_{1}$ > 1 and $DI_{2}$ > 1, the K indicator will exceed 1, and the defect is considered to be detected, so the threshold of the APCA algorithm is 1. Meanwhile, when the K index is used to evaluate a series of original signals and test signals, a damage index curve recording the changing trend of signals can be generated in the following experimental results.

## 3. Experiments and Results

### 3.1. Experimental Introduction

In this section, the guided wave signals of a straight pipe, bent pipe, and spiral pipe are presented, along with comparisons of the APCA, OBS, and the AED algorithm. The excitation parameters of the guided wave in several experiments are also reported.

In the first experiment, a straight pipe (aluminum) was used, and the semi-analytical finite element method (SAFE) was used to solve the dispersion curve [36,37]; the guided wave of T (0,1) mode with 128 kHz was selected for excitation because of its excellent propagation characteristics [38]. For the spiral pipe experiment, the early research of Zhang and Tang has proven that the comb magnetostrictive patch transducer (HCMPT) parallel to spiral weld [39] can effectively excite the pure bending T-mode guided wave in the spiral pipe (*f* = 64 kHz, diam = 0.72 m); under this condition, a superior echo waveform was recorded in this experiment. For the bent pipe experiment, because the SAFE method is only related to the cross-section shape and because Vinogradov’s Field testing on a carbon steel pipe indicated that higher frequencies (128 kHz) provided better performance in penetrating past U-bends [40], the same excitation conditions as those used for the straight pipe experiment were therefore used.

In the experiment, the transducer was fixed permanently in the process of collecting original signals and test signals, and the defect was judged by comparing test signals with the original signals collected on the same pipe. The transducer worked in the pulse-echo mode, which means it acted as both a guided wave transmitter and a guided wave receiver.

### 3.2. Straight Pipe Experiment

**Figure 10:**
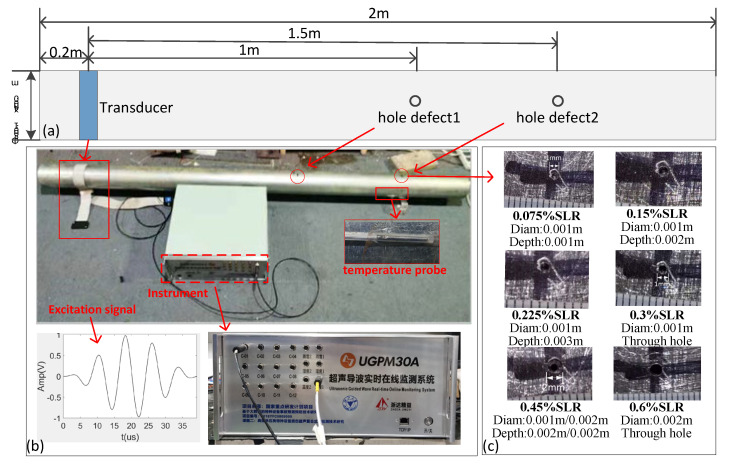
Straight pipe experiment conditions: (**a**) Pipe parameters; (**b**) Instrument and excitation signal (128 Khz); (**c**) Parameter of hole defects.

As shown in the straight pipe experiment conditions in Figure 10a, the transducer based on magnetostrictive effect [41] was installed 0.2 m from the beginning. A hole defect 1 m away from the transducer was expanded from small to large. As is shown in Figure 10c, the SLRs were 0.075%, 0.15%, 0.225%, 0.3%, 0.45%, and 0.6%.

The original signals of the first hole were collected in the pipe without any defects. After the first hole was expanded to an SLR of 0.6%, a second hole defect 1.5 m away from the transducer was produced in the same pipe. The original signals of the second hole were collected on the pipe with a hole. The guided wave was reflected at the first hole, which resulted in an energy reduction when the guided wave transmitted to the second hole.

The guided wave monitoring equipment (UGPM30A, Zheda Jingyi Tech, Ltd., Hangzhou, China) was used to acquire guided wave signals of the pipes. The excitation signal was a 128 kHz sinusoidal signal modulated by a Hanning window with a certain bandwidth, as shown in Figure 10b. The connection of temperature probe and transducer and the experimental site are also shown in Figure 10b. In this experiment, the original signals were collected every 10 min, and 100 sets of original signals were reserved. Similarly, 20 groups of test signals were collected for each kind of defect. We also collected 20 sets of signals before producing the hole for testing. See Table 1 and Table 2 for details.

In order to show the experimental results clearly, we calculated the recognition accuracy of the OBS, AED, and APCA methods on the same original signals and test signals. The accuracy is obtained by Equation (26):

(26) $$\mathrm{accuracy}=\frac{N_{judge}}{N_{actual}}\times 100\%$$

where the $\;N_{actual}$ means the number of actual defect samples, and the $N_{judge}$ means the number of defect samples judged by algorithm (when the threshold was exceeded). Similarly, the misjudgment rate of 0% SLR defect is obtained by Equation (27):

(27) $$\mathrm{error}=\frac{N_{judge}}{N_{actual}}\times 100\%$$

**Figure 11:**
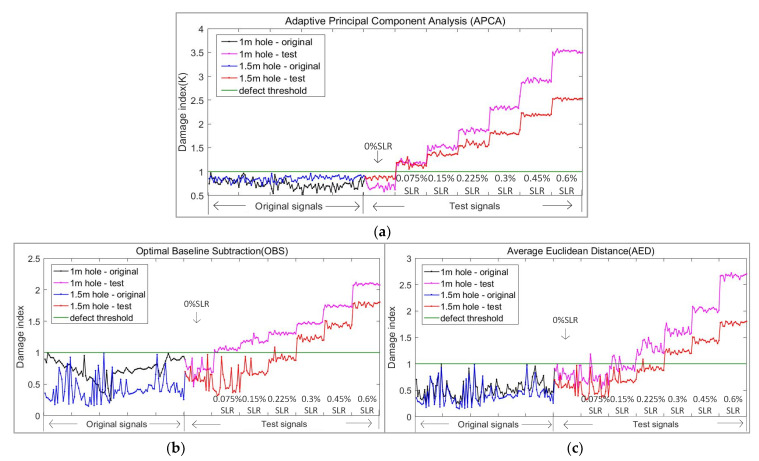
Damage index curve of different algorithms in straight pipe experiment: (**a**) Adaptive principal component analysis (APCA); (**b**) Optimal baseline subtraction (OBS); (**c**) Average Euclidean distance (AED).

The other two methods are the following:

- OBS: First, we calculated the mean value of the original signals, then we subtracted it from all the signals; the max value of the subtraction result was then taken to represent a sample. The threshold was the max value of the subtraction result of original signals. The damage index can be obtained by Equation (28):
  (28) $$\mathrm{damage}\;\mathrm{index}(\mathrm{OBS})=\frac{\max\left(x_{t}-\bar{x}\right)}{\max\left(\max\left(x_{i}-\bar{x}\right)\right)}$$
  where the $x_{t}$ means the original signals and test signals in the experiment, and the $x_{i}$ means the original signals. After the above processing, we can also obtain a damage index curve with a threshold of 1, as is shown in Figure 11b.
- AED: First, we calculated the mean value of the original signal, then we took the Euclidean distance between this and all signals. The damage index and threshold can be obtained by Equation (29):
  (29) $$\mathrm{damage}\;\mathrm{index}(\mathrm{AED})=\frac{\left(x_{t}-\bar{x}\right){\left(x_{t}-\bar{x}\right)}^{T}}{\max\left(\left(x_{i}-\bar{x}\right){\left(x_{i}-\bar{x}\right)}^{T}\right)}$$

After the above processing, we can also obtain a damage index curve with a threshold of 1, as is shown in Figure 11c. The experimental results are finally shown in Figure 11 and Table 3 and Table 4; each of them shows three methods.

As can be seen from the above Figure 11 and Table 3 and Table 4, for defects at 1 m, on the premise of 100% accurate identification, the minimum defect monitoring threshold of the APCA method was 0.075% SLR, the OBS was 0.15% SLR, and the AED was 0.225% SLR. For defects at 1.5 m, the minimum defect monitoring threshold of APCA was 0.075% SLR, and the OBS and the AED were 0.3% SLR. At the same time, the damage index curve obtained by APCA is smoother than that of OBS and AED.

From Figure 11, we can also see that the damage index of the defect at 1.5 m rises slower than the defect at 1 m; the guided wave is reflected at the 1 m hole, which also makes it more difficult to monitor the hole defect at 1.5 m.

It can be observed from Figure 11a that the instances and levels of the structural changes are clearly shown as step changes in the damage index curve of APCA; the threshold 1 obtained by the algorithm can also clearly distinguish the pipe without defect and the pipe with a defect of 0.075% SLR. At the same time, the algorithm also did not misjudge the signals without defect.

### 3.3. Spiral Pipe Experiment

**Figure 12:**
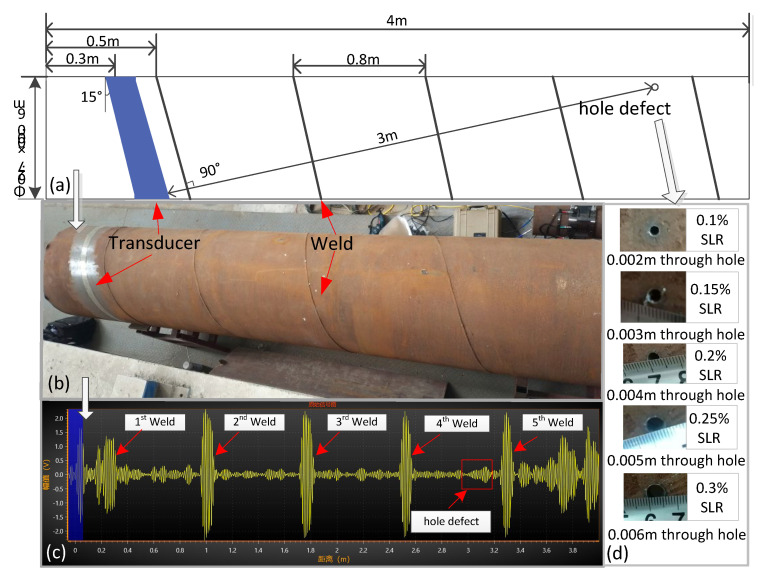
Spiral pipe experimental conditions: (**a**) Pipe parameters; (**b**) Pipe shape; (**c**) Instrument signal; (**d**) Parameter of hole defects.

Most of the experimental conditions have been mentioned above; however, a few conditions were different for this experiment. The experimental conditions for the spiral pipe (carbon steel) can be clearly seen in Figure 12. The transducer was installed at an angle of 15 degrees, parallel to the weld, and after another four welds, a through hole (0.1%, 0.15%, 0.2%, 0.25%, 0.3% SLRs) was produced 3 m away from the transducer.

The collection time and temperature of signals were basically the same as those in Table 1 in our laboratory. Finally, we obtained 100 groups of original signals without defects and 20 groups of five kinds of defects. We also collected 20 sets of signals before producing a hole for testing. It can be seen from Figure 12c that the characteristics of the spiral weld were well preserved. The difficulties of spiral pipe monitoring mainly lie in the following two points:

1. Due to the introduction of the weld, the guided wave echo amplitude of a defect is lower, and the signal-to-noise ratio will also be affected.
2. Compared with the straight pipe, the pure guided wave mode on the spiral pipe is more difficult to excite in the spiral pipe.

**Figure 13:**
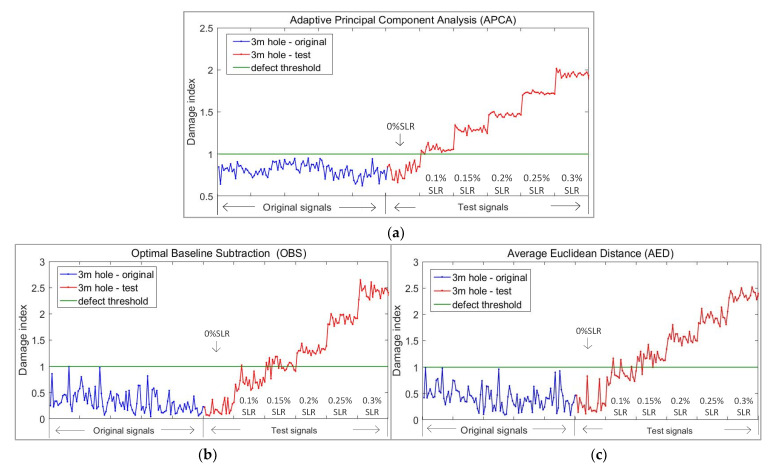
Damage index curve of different algorithms in spiral pipe experiment: (**a**) Adaptive principal component analysis (APCA); (**b**) Optimal baseline subtraction (OBS); (**c**) Average Euclidean distance (AED).

Similar to the straight pipe experiment, the final experimental results are shown in Figure 13 and Table 5.

As can be seen from the above Figure 13 and Table 5, on the premise of 100% accurate identification, the minimum defect monitoring threshold of the APCA method was 0.15% SLR, and the AED and OBS were 0.2% SLR. We can also find that the smoothness of damage index K in the spiral pipe was lower than that of the straight pipe, which shows that the performance of the APCA algorithm in spiral pipe is not as good as that in straight pipe.

### 3.4. Bent Pipe Experiment

**Figure 14:**
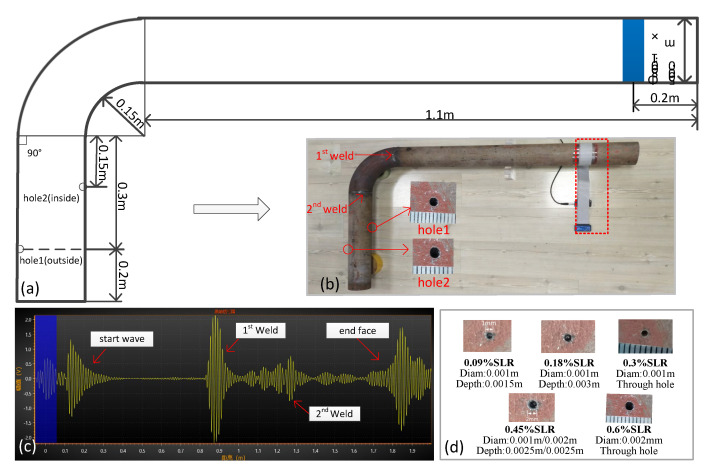
Bent pipe experiment conditions: (**a**) Pipe parameters; (**b**) Pipe shape; (**c**) Instrument signal; (**d**) Parameter of hole defects.

Again, most of the experimental conditions of the bent pipe have been mentioned above; however, there were just a few different conditions used. As shown in Figure 14a, the pipe (carbon steel) had two welds in the elbow area. The first hole was on the outside, and the second was on the inside. The two holes went from small to large, and the SLRs were 0.09%, 0.18%, 0.30%, 0.45%, and 0.6%.

The acquisition time and temperature of signals were basically the same as those in Table 1; we obtained 100 groups of original signals without defects and 20 groups of five kinds of defect. We also collected 20 sets of signals before producing hole for testing. The difficulties of bent pipe monitoring mainly lie in the following two points:

1. Multiple reflections occur between two welds when the guided wave propagates; moreover, the propagation of guided waves in the bend region is often accompanied by the mode conversion and dispersion.
2. The guided wave will focus on the outer surface of the elbow region, making it more difficult to monitor the inner surface [42].

The pipe and defect information is shown in Figure 14d. As we can see in Figure 14c, the guided wave signal contains many overlapping arrivals [43] and ringing in bend region, the SNR after the bend region is also lower. This makes it difficult to find defects intuitively from the waveform.

**Figure 15:**
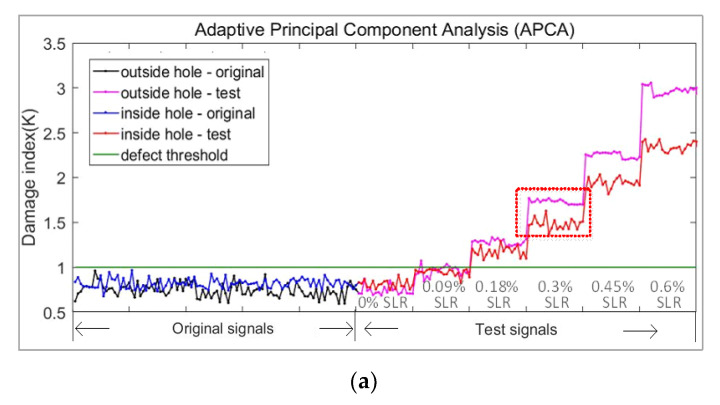

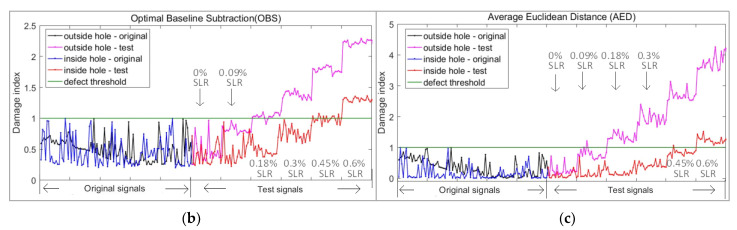
Damage index curve of different algorithms in bent pipe experiment: (**a**) Adaptive principal component analysis (APCA); (**b**) Optimal baseline subtraction (OBS); (**c**) Average Euclidean distance (AED).

The final experimental results are shown in Figure 15 and Table 6 and Table 7.

As can be seen from the above Figure 15 and Table 6 and Table 7, for defects on the outside of the bent pipe and on the premise of 100% accurate identification, the minimum defect monitoring threshold of APCA method was 0.18% SLR, the AED was 0.18% SLR, and the OBS was 0.3% SLR. For defects on the inside of the bent pipe, the minimum defect monitoring threshold of APCA was 0.18%, the OBS and the AED was 0.6% SLR. The results show that the inside defect was more difficult to monitor. At the same time, even at the same defect degree highlighted in the square box in Figure 15a, the damage index of outside defect was larger and smoother than that of inside defect.

### 3.5. Defect Localization

**Figure 16:**
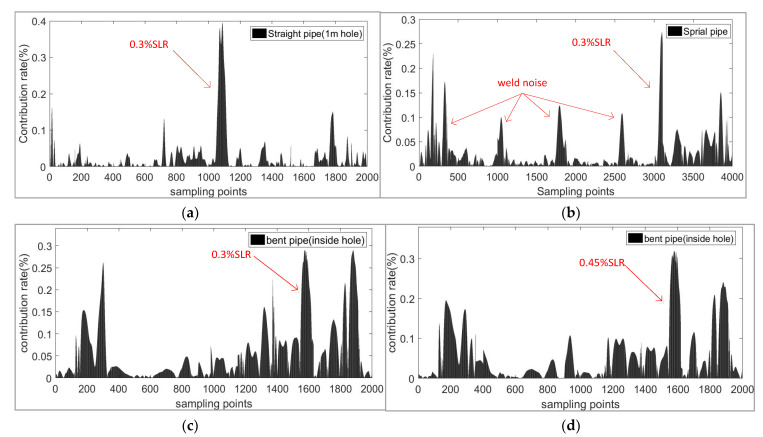
The contribution rate of sampling points in three experiments. (**a**) Hole defect of 0.3% cross section loss ratio (SLR) in straight pipe; (**b**) Hole defect of 0.3% SLR in spiral pipe; (**c**) Hole defect of 0.3% SLR in bent pipe; (**d**) Hole defect of 0.45% SLR in bent pipe.

In order to identify the location of defects, the defect information was determined by calculating the contribution rate of each sample point to $T^{2}$ and the SPE. The contribution rate of each sample ($CR_{j}$) was calculated as the following Equation (30):

(30) $$CR_{j}=\frac{x_{j}U_{kj}^{\;}\;S_{k}{}^{-1\;}U_{kj}^{T}\;x_{j}}{2*T^{2}\left(x\right)}+\frac{||U_{n-k\;j}^{T}\cdot x_{j}||{\;}^{2}}{2*SPE\left(x\right)}$$

where $CR_{j}$ is the contribution of a point in a sample to the result. The final contribution diagram is shown in Figure 16a; the straight pipe defect can be identified clearly in the range of 1000–1100 sampling points. The actual location distance of defect is calculated by the following Equation (31):

(31) $$L=\frac{1}{f_{s}}\times N\times v_{g}/2$$

where the $f_{s}$ means the sampling rate of the instrument, *N* means the sampling points, and $v_{g}$ means the speed of guided wave. The $v_{g}$ of T-mode guided wave in our experiment was about 3250 m/s according to the dispersion curve produced by Zhang [39], the time should be divided by 2 in a pulse echo experiment, and the $f_{s}$ was 1.625 Mhz in our experiment, so the instrument sampled 1000 points per meter—thus the 1000–1100 sampling points, corresponding to the actual distance of 1.0 m to 1.1 m.

Compared with the result of the spiral pipe, the SNR of the straight pipe was obviously higher, and Figure 16b shows that this was mainly due to the influence of the weld. Multiple reflections of guided waves occur between multiple welds and then lead to the overlapping arrivals of signal. At the same time, for the bent pipe, for signals of different defects in Figure 16c,d, the defect information of an SLR of 0.3% is not obvious, but when the SLR reached 0.45%, the defect’s contribution grew greatly, and we can deduce that the defect was located at 1500–1700 feature points.

## 4. Conclusions

This paper discusses an APCA method for the monitoring of pipe defects. We established an effective damage index by selecting the number of principal components adaptively and decomposing the characteristics of the original signals. Moreover, the contribution diagram given by the algorithm enriched the defect location information. The results of the above experiments demonstrate that the recognition sensitivity of the APCA method is higher than that of the OBS and AED methods. At the same time, the APCA method also did not misjudge the undamaged test signals in three experiments. In future research, our group will conduct on-site long-term experiments when conditions permit, so that the APCA-based method can be used more effectively for monitoring the health of pipes in service.

## Figures and Tables

**Table 1:** Sample signal information for the straight pipe (produce hole at 1 m).

Signals	Defects	Distance (m)	Number of Signals	Temperature (°C)
Original signals	Before producing hole (0% SLR)	/	100	21.4–24.6
Test signals	Before producing hole (0% SLR)	1	20	22.8–23.8
	Hole Defect 1 (0.075% SLR)	1	20	21.2–22.2
	Hole Defect 2 (0.15% SLR)	1	20	24.8–25.2
	Hole Defect 3 (0.225% SLR)	1	20	23.3–24.5
	Hole Defect 4 (0.3% SLR)	1	20	23.3–23.6
	Hole Defect 5 (0.45% SLR)	1	20	22.9–23.9
	Hole Defect 6 (0.6% SLR)	1	20	23.8–24.6

**Table 2:** Sample signal information for the straight pipe (produce hole at 1.5 m).

Signals	Defects	Distance (m)	Number of signals	Temperature (°C)
Original signals	Before producing hole (0% SLR)	/	100	20.8–24.4
Test signals	Before producing hole (0% SLR)	1.5	20	23.2–23.5
	Hole defect 1 (0.075% SLR)	1.5	20	23.5–24.3
	Hole Defect 2 (0.15% SLR)	1.5	20	23.5–24.1
	Hole Defect 3 (0.225% SLR)	1.5	20	23.8–24.6
	Hole Defect 4 (0.3% SLR)	1.5	20	22.2–22.8
	Hole Defect 5 (0.45% SLR)	1.5	20	22.4–23.6
	Hole Defect 6 (0.6% SLR)	1.5	20	22.4–23.0

**Table 3:** Comparison of algorithms in straight pipe (produce hole at 1 m).

Algorithm	0% SLR	0.075% SLR	0.15% SLR	0.225% SLR	0.3% SLR	0.45% SLR	0.6% SLR
	Error	Accuracy	Accuracy	Accuracy	Accuracy	Accuracy	Accuracy
APCA	0%	100%	100%	100%	100%	100%	100%
OBS	0%	90%	100%	100%	100%	100%	100%
AED	0%	5%	30%	100%	100%	100%	100%

**Table 4:** Comparison of algorithms in straight pipe (produce hole at 1.5 m).

Algorithm	0% SLR	0.075% SLR	0.15% SLR	0.225% SLR	0.3% SLR	0.45% SLR	0.6% SLR
	Error	Accuracy	Accuracy	Accuracy	Accuracy	Accuracy	Accuracy
APCA	0%	100%	100%	100%	100%	100%	100%
OBS	0%	0%	0%	5%	100%	100%	100%
AED	0%	0%	0%	5%	100%	100%	100%

**Table 5:** Comparison of algorithms in spiral pipe.

Algorithm	0% SLR	0.1% SLR	0.15% SLR	0.2% SLR	0.25% SLR	0.3% SLR
	Error	Accuracy	Accuracy	Accuracy	Accuracy	Accuracy
APCA	0%	95%	100%	100%	100%	100%
OBS	0%	5%	60%	100%	100%	100%
AED	0%	20%	90%	100%	100%	100%

**Table 6:** Comparison of algorithms in bent pipe with outside hole defect.

Algorithm	0% SLR	0.09% SLR	0.18% SLR	0.3% SLR	0.45% SLR	0.6% SLR
	Error	Accuracy	Accuracy	Accuracy	Accuracy	Accuracy
APCA	0%	5%	100%	100%	100%	100%
OBS	0%	0%	75%	100%	100%	100%
AED	0%	5%	100%	100%	100%	100%

**Table 7:** Comparison of algorithms in bent pipe with inside hole defect.

Algorithm	0% SLR	0.09% SLR	0.18% SLR	0.3% SLR	0.45% SLR	0.6% SLR
	Error	Accuracy	Accuracy	Accuracy	Accuracy	Accuracy
APCA	0%	0%	100%	100%	100%	100%
OBS	0%	0%	0%	0%	40%	100%
AED	0%	0%	0%	0%	5%	100%

## Data Availability

The original signals and test signals presented in this study are available on request from the corresponding author.
